# The CORE Service Improvement Programme for mental health crisis resolution teams: study protocol for a cluster-randomised controlled trial

**DOI:** 10.1186/s13063-016-1283-7

**Published:** 2016-03-22

**Authors:** Brynmor Lloyd-Evans, Kate Fullarton, Danielle Lamb, Elaine Johnston, Steve Onyett, David Osborn, Gareth Ambler, Louise Marston, Rachael Hunter, Oliver Mason, Claire Henderson, Nicky Goater, Sarah A. Sullivan, Kathleen Kelly, Richard Gray, Fiona Nolan, Stephen Pilling, Gary Bond, Sonia Johnson

**Affiliations:** Division of Psychiatry, UCL, 149 Tottenham Court Road, London, W1T 7NF UK; Onyett Entero Ltd, Care of University of the West of England, Health and Life Sciences Coldharbour Ln, Bristol, BS16 1QY UK; Department of Statistical Science, UCL, Gower Street, London, WC1E 6BT UK; Department of Primary Care and Population Health, UCL Medical School (Royal Free Campus), Rowland Hill Street, London, NW3 2PF UK; Department of Clinical Education and Health Psychology, UCL, Gower Street, London, WC1E 6BT UK; Institute of Psychiatry, Psychology and Neuroscience, Kings College London, 16 De Crespigny Park, London, SE5 8AF UK; West London Mental Health NHS Trust, Uxbridge Road, Southall, London, UB1 3EU UK; Epidemiology and Health Services Research, CLAHRC West, Lewins Mead, Bristol, BS1 2NT UK; Oxfordshire Healthcare NHS Foundation Trust, Barnes Unit, John Radcliffe Hospital, Oxford, OX3 9DU UK; Mental Health Sciences, University of the West of England, Coldharbour Lane, Bristol, BS16 1QY UK; Centre for Outcomes Research and Effectiveness, Division of Psychology and language Sciences, UCL, 1-19 Torrington Place, London, WC1E 7HB UK; Department of Psychiatry, Dartmouth Psychiatric Research Centre, Geisel School of Medicine at Dartmouth, Dartmouth-Hitchcock Medical Center, One Medical Center Drive, Lebanon, NH 03756 USA

**Keywords:** Crisis resolution teams, Acute care, Service improvement, Randomised controlled trial

## Abstract

**Background:**

As an alternative to hospital admission, crisis resolution teams (CRTs) provide intensive home treatment to people experiencing mental health crises. Trial evidence supports the effectiveness of the CRT model, but research suggests that the anticipated reductions in inpatient admissions and increased user satisfaction with acute care have been less than hoped for following the scaling up of CRTs nationally in England, as mandated by the National Health Service (NHS) Plan in 2000. The organisation and service delivery of the CRTs vary substantially. This may reflect the lack of a fully specified CRT model and the resources to enhance team model fidelity and to improve service quality. We will evaluate the impact of a CRT service improvement programme over a 1-year period on the service users’ experiences of care, service use, staff well-being, and team model fidelity.

**Methods/design:**

Twenty-five CRTs from eight NHS Trusts across England will be recruited to this cluster-randomised trial: 15 CRTs will be randomised to receive the service improvement programme over a 1-year period, and ten CRTs will not receive the programme. Data will be collected from 15 service users and all clinical staff from each participating CRT at baseline and at the end of the intervention. Service use data will be collected from the services’ electronic records systems for two 6-month periods: the period preceding and the period during months 7-12 of the intervention. The study’s primary outcome is service user satisfaction with CRT care, measured using a client satisfaction questionnaire. Secondary outcomes include the following: perceived continuity of care, hospital admission rates and bed use, rates of readmission to acute care following CRT support, staff morale, job satisfaction, and general health. The adherence of the services to a model of best practice will be assessed at baseline and follow-up. Outcomes will be compared between the intervention and control teams, adjusting for baseline differences and participant characteristics using linear random effects modelling. Qualitative investigations with participating CRT managers and staff and programme facilitators will explore the experiences of the service improvement programme.

**Discussion:**

Our trial will show whether a theoretically underpinned and clearly defined package of resources are effective in supporting service improvement and improving outcomes for mental health crisis resolution teams.

**Trial registration:**

Current Controlled Trials ISRCTN47185233

**Electronic supplementary material:**

The online version of this article (doi:10.1186/s13063-016-1283-7) contains supplementary material, which is available to authorized users.

## Background

Crisis resolution teams (CRTs) – otherwise referred to as home treatment or crisis assessment teams – provide rapid assessment in mental health crises and offer intensive home treatment as an alternative to acute admission if feasible [1]. The introduction of CRTs, mandated by the National Health Service (NHS) Plan in 2000 [2], has been an extensive change in the English mental health care system. In 2000, few areas had such teams, but at the time of writing, CRTs are available in every Trust in the country, and several thousand mental health professionals have migrated into them [3]. UK government guidance [1] recommended that CRTs should provide an easy access, rapid response, 24-hour service; should be multi-disciplinary and able to provide medical, psychological, and social interventions; and should help facilitate early discharge and adopt a ‘gatekeeping’ function of assessing all service users before admission to acute wards to ensure home treatment is presented as an alternative to admission wherever possible [4]. When CRTs first became national policy, their evidence base was criticised as limited [5, 6]. However, some positive findings have now been reported from naturalistic studies and a randomised controlled trial, suggesting that CRTs reduce inpatient admissions [7–11] and healthcare costs [12, 13] and increase service user satisfaction with acute care [7, 10].

Despite indications of the potential effectiveness of CRTs, considerable reservations have emerged about their delivery in routine settings in the UK, most notably in recent reports by Mind [14], the Schizophrenia Commission [15], and the Care Quality Commission [16]. Both CRT and ward managers still view a significant minority of hospital admissions as unnecessary [17]. The impact on bed use appears to vary considerably between areas [11, 18, 19], and the reductions in bed days tend to be less marked than those in admissions [9, 11]. Rates of compulsory admissions have continued to rise in England since 2000 despite the national implementation of CRTs [18–20], and a recent national audit raised concerns about the number of suicides by service users receiving CRT care [21]. Service users and carers, whilst mainly positive about receiving care in their own homes, report important areas of dissatisfaction with CRTs [14, 22, 23], especially regarding the continuity of care, the quality of relationships with staff, and a narrow range of support on offer, which sometimes focuses principally on medication and short-term symptom control.

The UK experience, and similar challenges in Norway, where CRTs are also mandated at the national level but with variable implementation [24], illustrates that the national mandate, policy, and guidelines have been insufficient to ensure consistent and complete implementation of this complex intervention. A survey of CRTs in 2005/6 [25] and another in 2011/12 [26] confirmed considerable variation in CRT resources, organisation, and service delivery. One potential reason for inconsistencies in CRT implementation is that the CRT model and its theoretical basis were not highly specified at the outset [4, 26], nor has a means of measuring fidelity to a model of good practice been developed and disseminated. Improving acute mental health services is a current UK policy priority [27], yet available quality improvement resources are lacking for CRTs to promote best practice.

The US National Implementing Evidence-Based Practices Project (EBP) [28, 29] offers a model for evaluating and promoting quality improvement in complex service-level mental health interventions. Integral elements of the EBP approach are service reviews using a fidelity measure that assesses how far services are achieving a model of good practice and utilisation of a resource kit to support implementation and quality improvement [30]. Resource kits typically consist of guidance, training materials, and coaching and support for service managers and staff, designed to help services address areas where high model fidelity has not been achieved. Modelling of factors associated with successful implementation of evidence-based practices has helped to theoretically underpin the EBP implementation model [31, 32]. Attention to workforce development and training may in itself be insufficient to achieve high fidelity implementation of complex mental health interventions: securing leadership support, organising workflow (service structures and organisation) to support implementation, and providing feedback to reinforce implementation successes are also required [31]. Access to technical assistance that enables understanding of the specific requirements of a complex intervention can also facilitate implementation [32]. The EBP programme has successfully developed fidelity scales and implementation resources for a range of service-level interventions including supported employment [33] and assertive community treatment [34]. Previous studies have found correlations between fidelity to an evidence-based practice and better client outcomes [35, 36]. CRTs are comparable with models in the EBP project because trial evidence exists for their efficacy in the right conditions. A CRT fidelity scale was developed in an earlier part of this programme of research (The CORE Study) [37]. This trial will test the effectiveness of the CORE CRT Service Improvement Programme in helping CRTs improve outcomes and achieve high model fidelity.

## Aims

This trial tests the CORE CRT Service Improvement Programme in a cluster-randomised trial. CRTs randomised to the intervention arm will receive the Service Improvement Programme over a 1-year period, and control CRTs will not receive the intervention. The study aims to investigate whether a CRT Service Improvement Programme can improve service users’ experience of CRT care, reduce acute service use, and improve CRT staff well-being. The primary outcome is service user satisfaction, measured using The Client Satisfaction Questionnaire (CSQ-8) [38]. We will also explore whether the fidelity scores of the CRT teams receiving the Service Improvement Programme rise over the 1-year intervention period and the associations between team fidelity score and service outcomes. Through a qualitative and process evaluation, we will seek to understand stakeholder experience of the CRT Service Improvement Programme, contextual factors constituting barriers and facilitators to its implementation, and any mechanisms by which it may improve team effectiveness.

## Methods/design

This cluster-randomised controlled trial is funded by the National Institute of Health Research (NIHR) and has received ethical approval from the Camden & Islington Research Ethics Committee (Ref: 14/LO/0107). It is registered on the ISRCTN registry (Ref: 47185233). The protocol reported in this paper corresponds to the current, ethically approved, version of the trial protocol: V3_25/06/15.

### Preliminary work

The lack of a highly specified model for CRTs [4, 26] led to the development of a fidelity scale by the CORE study team [37]. Fidelity refers to adherence to the standards defining a specific practice; a fidelity scale is a quantitative measure assessing the degree of adherence. The fidelity scale was developed from qualitative interviews with stakeholders [39], a systematic review of previous research [40], and a national survey of CRT managers [26]. The CORE CRT fidelity scale defines 39 fidelity criteria; each item is scored on a scale of 1–5, with 5 indicating excellent fidelity and 1 very low fidelity; and a total score is yielded that ranges from 39–195. The scale has been piloted and used to survey CRT model fidelity across 75 CRT teams in England in 1-day reviews by three trained external reviewers. Whilst completing the 75 team fidelity surveys, researchers collected best practice resources and case study examples that inform the development of the Service Improvement Programme, which is designed to help CRT services achieve high model fidelity and quality improvement.

### Main study

The CORE Trial involves the collection of data from 25 CRTs at baseline and at the 1-year follow-up. The trial comprises four components: (1) quantitative evaluations of service user experience, (2) quantitative evaluations of service use, 3) quantitative evaluations of staff well-being, and 4) an accompanying qualitative and process evaluation. This protocol paper follows the SPIRIT recommendations [41] for trial protocols. A copy of the SPIRIT checklist, detailing where each recommended element of protocol reporting is included in this protocol paper, is provided as Additional file 1.

### Setting

Twenty-five CRTs will be recruited to the trial. CRTs will be selected from NHS Trusts in four regions of England (North London, South London and the South-east, the West of England, and the heart of England) to reflect a range of Trusts and urban and more rural areas. We excluded the CRT teams identified by our earlier CORE fidelity survey as already achieving good model fidelity (defined as having a mean item score of 4 or higher). Fifteen CRT teams were included in the intervention arm of the trial to allow a thorough investigation of the implementation of the intervention in a range of contexts. Ten teams were included in the control group, as this was considered sufficient to represent treatment as usual. The number of participants in each outcome is therefore greater in the intervention than the control arm, although it is equal within each cluster for the primary outcome.

### Randomisation

The 25 teams will be randomised to either receive the Service Improvement Programme (n = 15) or the control (n = 10) after the baseline fidelity reviews have taken place for all participating teams within each NHS Trust. Randomisation of the CRTs will be stratified by the NHS Trust to ensure that within each Trust, some CRTs will receive the intervention and some will act as control teams, to address the potential confounding factor of Trusts’ macro-level management and service processes. Randomisation will be conducted by a Priment (a University College London Clinical Trials Unit) statistician who is independent of the study. Randomisations will be conducted for all participating teams within each NHS Trust at the same time, once baseline fidelity reviews have been completed at all sites within the Trust. The CORE trial has made no arrangements for blinding: both participating services and researchers will be aware of the team allocation status. Service user participants providing data for the trial’s primary outcome are not expected to be informed of their team allocation, and the NHS Informatics Teams providing anonymised patient service-use data will not be aware of the team allocation status.

## Sample

The four components to this study are presented and described separately.

### 1) Service user experience

At each CRT, 15 service user participants will be recruited at baseline, and 15 service user participants will be recruited who were discharged in months 10–12 of the 12-month intervention, providing a total sample of N = 375 at each time point. At each service, we will screen and recruit consecutively discharged, eligible, and consenting service users until we reach our target of 15. Eligibility criteria for participants are the use of the CRT service for at least 7 days, ability to read and understand English, capacity to provide informed consent, and do not pose too high a risk to others to participate (including being interviewed on NHS premises or participating by phone, email, or online survey).

### 2) Service use

Anonymised service data for two cohorts of service users will be collected at two time points – baseline and follow-up: (1) a cohort of all service users admitted to the CRT during a 1-month period ending 6 months prior to the study baseline date and another cohort of all service users admitted during month 7 of the study intervention period at each Trust and (2) all service users admitted to the acute inpatient services during a 6-month period up to the study baseline date and during months 7–12 of the study intervention period at each Trust.

### 3) Staff well-being

At each CRT, all clinical staff will be invited to complete a set of questionnaires at baseline and outcome (months 10–12) time points to measure staff morale and psychological health.

### 4) Qualitative and process evaluation

#### Fidelity reviews

Fidelity reviews will involve individual interviews with CRT managers and a separate focus group with available CRT clinical staff. The CRT team will invite discharged service users (n = 6) and carers (n = 6) to take part in short phone interviews. Managers of other community teams (n = 3), who make referrals to the CRT and ward managers (n = 2) who work with the CRT, will be interviewed. Anonymised case notes of recently discharged service users (n = 10) and CRT routine records, policies, and protocols will be reviewed.

### Facilitator monthly updates

The CRT service improvement facilitators (n = 7) will be interviewed each month throughout the 12-month intervention by a member of the research team who will record the implementation activities undertaken by each team (n = 15). Interviews will be conducted with the ten control team managers at 6 months and the end of the study period to check for possible contamination, i.e. take up of service improvement initiatives or shared learning from intervention teams within the same Trust.

### Qualitative Evaluation

Six case study sites will be purposively selected following 12-month fidelity reviews to include teams in urban and more rural settings, teams starting from comparatively high and low baseline model fidelity, and teams where an improvement in the fidelity score was achieved and where it was not. Interviews with CRT manager and a separate focus group with up to ten CRT team members per team will be conducted at each case study site. We will seek to include staff representing a range of professional groups, levels of seniority and amount of CRT experience within each focus group. Interviews will also be completed with all CRT facilitators (n = 7) and their clinical supervisor (n = 1).

### Sample size

A sample size calculation for the primary outcome measure (service user satisfaction measured using the Client Satisfaction Questionnaire [38]) determined the size of the service user sample. A sample of 375 participants (225 from 15 CRTs that have implemented the service improvement intervention and 150 from ten CRTs that have not) will give 97 % power to detect half a standard deviation difference in mean satisfaction (3.5 points assuming a typical SD of 7.0), and 80 % power to detect a small difference of just over one-third of a standard deviation, allowing for moderately large within-team clustering (ICC = 0.05).

## The intervention

The CORE CRT Service Improvement Programme is designed to support teams to identify target areas for service improvement and to produce and implement plans that improve current practice. Structures and resources included in the Service Improvement Programme are available in the online CRT Resource Pack. They include the following:

1. Assessment of adherence to current CRT best practice measured by the CORE CRT fidelity scale. Fidelity reviews will be conducted at baseline and at the end of the 12-month study period with control teams and intervention teams. Intervention teams will receive an additional 6-month review, with feedback from the external reviewers being included on the resulting fidelity report to the CRT manager and team. Intervention teams will be offered a meeting with the reviewers to discuss their 6-month report. This will be used to identify targets for service improvement and planning for how to achieve them and to provide positive reinforcement of any implementation successes achieved during the previous 6 months.
2. Structures to guide service improvement work. A number of structures based on an EBP framework will be utilised to guide service improvement. These structures include a 1-day, whole-team scoping event for each CRT to kick start the Service Improvement Programme and feedback on the fidelity review; service improvement groups (SIGs) of managers and clinical leaders within each CRT meeting regularly to develop service improvement plans (SIPs); and collaboration between CRT managers and staff in teams receiving the intervention, which will be promoted by the research team. Collaboration activities will include an online forum; regular bulletins from the research team about implementation progress at study sites; and at least two meetings/events during the study period to promote sharing of experience, knowledge, and best practice. Evidence suggests that these types of collaborative learning events have the potential to support improvements in the quality of services [42].
3. A local facilitator. Participating NHS Trusts will fund a local facilitator with dedicated time (0.1 full time equivalent for each intervention team) to help teams implement the Service Improvement Programme by encouraging the use of the resource pack; discussion and coaching of the CRT manager; mentoring, supervision, and training of the CRT staff; and liaison with senior Trust management regarding resources or organisational support required to achieve model fidelity. The local facilitator may either be an employee of the participating NHS Trust or an external consultant identified by the study team, depending on local resources and preferences. The facilitator will typically be a manager or senior clinician with experience of working in or with CRTs. Facilitators will be provided with initial and follow-up group training and individual coaching at least once a month by appropriately experienced members of the research study team (a clinical psychologist with CRT clinical experience and a clinical and academic psychologist with expertise in leadership and change management in mental health service contexts). Facilitators will be invited to regular implementation meetings with the study team to offer feedback on how the intervention is progressing and discuss ways of overcoming barriers to service improvement.
4. Access to a web-based resource pack manual, including practical resources, guidance, and training materials to support implementation; case studies from high fidelity CRT services outlining strategies for achieving high fidelity; relevant reading; useful links; video and audio clips of service users, carers, and staff involved in clinical CRT work; and service improvement research providing rationale for fidelity scale items and ideas for improving support. An outline of the structures designed to help facilitate service improvement over the 1-year intervention will also be included.

The CORE CRT Service Improvement Programme, with ongoing implementation support from facilitators during the year of the intervention, will thus provide ongoing help to teams during the course of the project in securing leadership support, structuring team workflows to support implementation, and providing expert technical assistance with implementing the CRT model where required, in line with EBP implementation theory [31]. Services in the control group will receive a fidelity review and written report at baseline and at the end of the study period but no other implementation support. Control teams will not be provided with details of the online resource pack; teams within the intervention group will undertake not to share resources or promote service improvement in the control teams until the end of the trial.

## Measures

### 1. Service user experience

Schedules will include information about service user characteristics and service use (age, gender, ethnicity, and previous use of the CRT and inpatient admissions). It will also include two structured measures:

- The Client Satisfaction Questionnaire (CSQ-8) [38] is an eight-item measure of satisfaction with the CRT service. Each item is scored on a four-point Likert-like scale, yielding a total score between 8 and 32 (high score = more satisfied). The CSQ-8 is a well-validated measure of service user satisfaction, which has demonstrated high reliability and validity, including in mental health service settings, and has high internal consistency (coefficient α = .91, and median item-total correlation value = .64).
- Continu-um [43] is a measure consisting of 16 topics relating to the perceived continuity of care, with responses each noted on a five-point Likert scale, scored 1 to 5, giving a possible range of 16 to 80, with higher scores indicating greater continuity of care.

### 2. Service use

Data on acute service use will be collected for two cohorts of service users at baseline and follow-up.

Cohort 1: Readmissions to acute care, compulsory and voluntary hospital admissions, and days with the CRT and in acute care during a 6-month follow-up period will be collected for service users admitted to the CRT during a 1-month period ending 6 months prior to the study baseline and for a second 1-month period in months 6–7 of the intervention period.

Cohort 2: The number of hospital admissions and inpatient bed-uses and the available summary demographic data for all service users within the CRT’s catchment area will be collected for service users admitted to acute inpatient services during a 6-month period up to the study baseline and for a second 6-month period during months 7–12 of the study intervention in each Trust.

### 3. Staff well-being

All CRT clinical staff will be asked to complete a self-report questionnaire at two time points: study baseline and 1-year follow-up. It will cover the demographic characteristics and the role of the CRT and will include the following:

- The Maslach Burnout Inventory [44]. This is a 22-item measure of staff morale, providing information about emotional exhaustion, cynicism, and perceived personal accomplishment. Its possible range is 0 to 132.
- The General Health Questionnaire [45]. This is a 12-item measure of general psychological health that yields a score ranging from 0 to 36.
- The Work-Related Acceptance and Action Questionnaire [46]. This is a seven-item scale of work-related psychological flexibility that yields a score ranging from 7 to 49.
- The Work Engagement Scale [47]. This is a nine-item measure of positive work engagement that yields a score ranging from 0 to 54.

### 4. Qualitative and process evaluation

#### Fidelity reviews

CRT model fidelity will be assessed in all teams at baseline and 1-year follow-up using the CORE CRT Fidelity Scale [37], a 39-item measure yielding a total score ranging from 39–95.

### Monthly process monitoring

Monthly process monitoring for each CRT in the intervention arm will identify the fidelity items targeted in the service improvement plans and categorise the CRT facilitators’ implementation activities using a typology developed by the EBP Program as prioritisation, leadership, workforce, workflow, or reinforcement [31] to describe the facilitators’ activities and explore how they may relate to changes in team fidelity during the project.

#### Qualitative interviews

Topic guides for local facilitator interviews and CRT manager interviews and staff focus groups will examine the participants’ experiences of the CRT service improvement intervention; most and least helpful parts of the intervention; barriers and facilitators to its implementation, including local contextual factors; and the perceived impact of the intervention implementation on the CRT service delivery and outcomes.

## Procedures

The 1-year study intervention period will be the same for all teams within each participating NHS Trust, defined as starting from the date when the Trust’s CRT facilitator starts in post. Outcome data from Trust patient records will be collected for a 6-month period 6–12 months following the intervention start date. Outcome interviews with service users and staff, and the end-of-study CRT fidelity review, will be conducted between months 10 and 12 of the trial-intervention period.

### 1) Service user experience

#### Screening and recruitment

Clinical staff in participating CRTs will be asked to screen and identify consecutively discharged potential service user participants who meet the study’s inclusion criteria. Eligible service users will be approached close to the point of discharge by clinical staff from the CRT that supports them, who will explain the study briefly and ask if they are willing to be contacted by a researcher. A study researcher will then contact potential participants to explain what the study involves, answer any questions, and send a written study information sheet [48]. The researcher will make contact again to check the participant has understood the information sheet and has continued capacity to consent. Consent to participate and completion of the questionnaire can be done in one of the following ways: at a face-to-face meeting, via post, over the telephone (consent will be audio-recorded and stored securely), or online via a link sent by researcher (using UCL’s secure “Opinio” system). A copy of the consent form is provided as Additional file 2.

The interview will take approximately 15 minutes to complete. This process will continue until 15 service users from each team have completed the questionnaire. Participating service users will be offered a gift of £10 in acknowledgement of their time, given as cash or as an e-voucher, according to the participant preference.

### 2) Service use

For baseline and study outcome data from patient records, a study researcher will contact the appropriate administrators or informatics team within each NHS Trust. The study researchers will provide a pro forma that specifies clearly the nature of the information and time periods for which data are required. Administrators will be asked to provide the required patient-level data in anonymised form.

### 3) Staff well-being

A study researcher will visit the CRT team to publicise the study and answer any questions the staff may have about their involvement. The study researcher will assign a study identification number to each staff member. A master document linking CRT staff names to ID numbers will be stored securely at the research study office. An email will then be sent to all CRT staff containing an invitation to participate in the study, a study information sheet, their individual ID number, and a link to the online structured questionnaire hosted on the UCL's secure network. Consent to participate in the study will be provided by staff through completing the questionnaire.

### 4) Qualitative and process evaluation

#### Qualitative interviews

At the end of the 12-month intervention, a study researcher will contact all local facilitators and the managers of the six case study CRTs directly to invite them to participate in an individual interview. Staff focus group participants will be identified initially through liaison with managers of case study CRTs. A study researcher will provide potential participants with written information about the study, and the focus group and will include information on how to contact the researchers with any questions about participating. Staff will be informed that participation is entirely voluntary. Written consent will be taken from participants before the focus groups begin. Focus groups will be facilitated by two researchers from the study team. Focus groups and individual interviews will be audio-recorded.

#### Process monitoring

All monthly monitoring data collected from progress update phone calls with local facilitators will be coded under the EBP implementation categories by a researcher each month. [31]. Usage of the online resource kit will be monitored using Google analytics.

In addition, phone calls will be made to control team managers at 6 and 12 months: any reported impact of the Service Improvement Programme on their own team’s practice and any other major service improvement initiatives affecting CRTs taking place within NHS Trusts will be recorded.

### Data management

Study researchers will develop and manage a secure database for all quantitative study data using SPSS software and will store electronic copies of focus group transcripts using Nvivo qualitative software [Nvivo9: http://www.qsrinternational.com/product ] on the secure IT network at University College London. The study team will follow advice from Priment, a UCL Clinical Trials Unit, regarding development and maintenance of the study database.

Staff data will be entered by staff themselves, via the secure UCL online questionnaire. Service user data will be entered either by participants themselves via the secure UCL online questionnaire, or by researchers after a phone or face-to-face interview, or receipt of a paper copy of the questionnaire. All data will be transferred to the trial SPSS databases by study researchers. The study Chief Investigator will act as custodian of the data.

### Study oversight

The study sponsors, the Camden and Islington NHS Foundation Trust, act as guarantors for the trial, including insurance and indemnity arrangements, and are responsible for overseeing and auditing trial conduct. The study is supported by the Priment Clinical Trials Unit at University College London. Management of the study is coordinated through a trial management group, consisting of the study chief investigator and trial manager, trial statisticians and health economist, senior investigators, and representatives of Priment. Any proposed changes to the trial protocol during the study will be agreed on by the trial management group and submitted for approval to the research ethics committee by the study team. Service user and staff participants providing outcomes data for the trial will all provide informed consent to take part, using ethically approved procedures. Independent advice to the study team and oversight of the study is provided by a trial steering committee, which is independent of the sponsor and will meet at least annually during the trial. The steering committee comprises senior academics, including a statistician and a health economist; clinicians with relevant experience in acute care and/or service improvement; and service user and carer representatives with expertise by experience. A data monitoring committee (DMC) is not planned for this team-level intervention, but the trial steering committee will advise if any role for a separate DMC is indicated during the trial. No interim analyses are planned, and no stopping criteria are pre-set. Any serious adverse events reported to the study team will be screened by the chair of the trial steering committee as an independent reviewer. Any adverse events assessed as study-related will be reported, with the trial steering committee chair’s recommendation, to the study sponsor and the research ethics committee. Annual study progress reports will be provided by the study team to the sponsor and the research ethics committee.

## Analysis

### 1) Service user experience

We will test the hypothesis that participant satisfaction with the crisis resolution team, measured by the client satisfaction questionnaire [38] is greater in the teams that have implemented the Service Improvement Programme than control teams. This will be analysed using a multivariate linear random effects model with a random effect for CRT (mixed model), controlling for the mean baseline client satisfaction questionnaire score by CRT. Experiences of continuity of care, measured using Continu-um [43], will also be compared between those receiving and those not receiving the intervention.

Second, we will explore, using random effects modelling with a random effect for CRT, the extent to which the team fidelity score can explain variations in individual satisfaction with care.

### 2) Service use

We will use routine data to compare service use patterns between teams that have received the Service Improvement Programme and the control teams. Data on admission rate, bed use, and population size will be measured over 6-month periods before and after intervention introduction. We will also explore whether there is any evidence of differences between intervention and control areas in extent of change in rates of compulsory detention under the Mental Health Act and of readmissions within 6 months of an initial admission to acute care. Other routinely collected indicators of CRT functioning, such as referral sources and caseload composition will also be examined. Data will be analysed using Poisson random effects modelling. Either bed use or admission rate will be set as the exposure variable as appropriate.

If differences between the two groups are found, national reference costs will be applied to resource use (admission, bed days and re admissions) to calculate the cost difference between resource kit implementation and control areas. A difference in difference model will be used, looking at before and after effects in the two areas using the most appropriate statistical model as determined by Akaike information criteria (AIC) and Bayesian information criteria (BIC) values. This is likely to be a general linear model with log link and family gamma. The model will take the same form as the Poisson model above.

### 3) Staff well-being

We will test the hypotheses that the mean staff psychological well-being scores, measured by the General Health Questionnaire [45], and the mean staff burnout scores, measured by the Maslach Burnout Inventory [44], will be lower in CRTs receiving the Service Improvement Programme and that the mean staff job involvement scores, measured by the Work Engagement Scale [47], will be higher in CRTs receiving the Service Improvement Programme. We will use data collected from CRT staff at study baseline before the introduction of the programme to assess whether there are baseline differences between the two groups of teams for which adjustments should be made.

In secondary analyses, we will explore whether staff psychological flexibility scores at baseline, measured using the Work-Related Acceptance and Action Questionnaire [46], predicts staff morale and job satisfaction following implementation of the Service Improvement Programme and whether psychological flexibility scores change following implementation.

### 4) Qualitative and process evaluation

#### Process data

1. Fidelity reviews: We will provide descriptive data regarding changes in the team fidelity scores over the 1-year study period. We will explore the extent to which team fidelity score can explain variations in individual satisfaction with care. This analysis will use linear random effects modelling, with a random effect for CRT.
2. Descriptive data on the number of implementation activities at each site and the proportion of total implementation activities of each type will be reported for each CRT team.
3. We will also report whether the following implementation structures recommended as part of the Service Improvement Programme were achieved at each intervention CRT: scoping day; development of a service improvement plan; formation of a service improvement group; whether the SIG met at least six times during the study; attendance of team/Trust representatives at a learning collaborative day.
4. For all 25 teams, we will report whether any other major service improvement initiatives took place during the study intervention year.
5. The online resource kit used by the CRT staff will be monitored via Google Analytics, which captures time spent on individual pages and document downloads.

#### Staff focus groups

Qualitative data from interviews with local facilitators and focus groups with staff from CRTs implementing the resource kit will be analysed using thematic analysis [49] aided by qualitative analysis software (Nvivo9). Thematic analysis will allow exploration of themes relating directly to our research questions and arising more inductively from the data. Analyses will be conducted collaboratively by a group of researchers within the team to enhance the validity of the analysis.

### Dissemination and access to trial results

The trial findings will be publicly available via a final report to the study funder, the National Institute for Health Research. The trial will also be reported in peer-reviewed journals. Information regarding the study, including the trial protocol, are available through the study website [50], and the web-based resource pack is now publicly available [51].

## Discussion

### Strengths

Strengths of the CORE phase 4 study include the following:

1. A multi-site, cluster-randomised trial will provide quality evidence regarding the effectiveness of the CORE CRT Service Improvement Programme. The wide spread of the teams involved will produce generalizable evidence regarding the effectiveness of the programme in a range of contexts. Evidence will be gathered on the relationship between fidelity to a clearly defined model of best practice and service outcomes.
2. The Service Improvement Programme follows a developed US evidence-based practice programme template for achieving high-fidelity implementation of a complex intervention or service model. Process monitoring will confirm whether key elements of the EBP approach are delivered in each team.
3. The mixed methods approach involving measurement of changes in team fidelity, process data, and qualitative evaluation will help to understand trial outcomes, mechanisms of change and barriers and facilitators to implementation and contextual factors that may influence the effectiveness of the trial intervention.
4. The trial outcome measures will allow evaluation of a number of important aspects of CRT services. Service users’ experience of care, measured using the Client Satisfaction Questionnaire [38], is the primary study outcome. The sample size in the trial is sufficient to detect a medium or large difference in satisfaction between participants in the intervention and control arm teams. The experience of care is inherently important in all health services research, and improving the service users’ experience is an overarching goal of the CORE CRT Service Improvement Programme. Service users’ experience of CRT care is especially important to evaluate in our trial, given recent reports of high levels of dissatisfaction with CRT and acute care in the UK [14, 16]. The trial will also investigate the impact of the CORE Service Improvement Programme on other outcomes relevant to the services’ clinical and cost effectiveness, i.e. local admission rates and service users’ recovery following CRT care, and will measure any impact of the team-level intervention on CRT staff well-being.

### Limitations

Limitations of the study relate to the following:

1. Blinding: Due to the need for researchers to have ongoing contact with CRT teams and facilitators throughout the intervention, it will not be possible for researchers to be blinded to team randomisation, and this approach could lead to researcher bias.
2. Contamination: As NHS Trusts often try to ensure their services are operating in a consistent way, a limitation of the study is the potential for contamination between teams receiving the Service Improvement Programme and control teams within the same Trust. To minimise this, researchers will ensure teams are informed that for the clarity of study findings it is beneficial if any CORE-related service improvement work is limited to the intervention teams until the end of the study, and this includes limiting access to the online resource kit. In addition, monitoring of contamination through interviewing control team managers will serve to highlight the extent of this.
3. Retrospective collection of some data: It will not be feasible to complete all baseline data collection before randomisation. However, all staff data will be collected before the scoping day – the kick-off, one-day team meeting in each CRT which is arranged as soon as possible after the start of the intervention year, and service user data will be collected from service users discharged before the scoping day. Due to the nature of the service user sample, different service users will be participating at baseline and follow-up.

## Implications for policy and practice

1. If the trial yields positive results, the CORE CRT Service Improvement Programme will provide a clearly defined structure and set of resources that have been demonstrated to help improve the quality of CRTs.
2. Regardless of the results of the trial concerning aspects of the whole CORE CRT Service Improvement Programme, the online resource kit will provide an ongoing training and service development resource to support implementation in CRTs, which provides practical implementation tips and training materials and best practice case examples.
3. The use of the CORE CRT fidelity scale as a valid tool to support service improvement and assess service quality will be supported in the event of exploratory analyses from the trial, indicating a relationship between team fidelity scores and outcomes.

## Research implications

The main trial results will provide preliminary evidence regarding whether the CORE CRT Service Improvement Programme is effective in increasing model fidelity and improving outcomes in CRTs. The study will also provide the following:

1. Evidence regarding the validity of the CORE CRT fidelity scale. We will explore, through multilevel modelling, whether team fidelity scores relate to important service user and staff outcomes.
2. Evidence regarding the feasibility of implementing the intervention from process data and barriers and facilitators to implementation from the qualitative evaluation, which can help understand any mechanisms of change and inform future implementation programmes.

Implementation represents the biggest challenge to translating research knowledge into patient benefit [52]. CRTs exemplify this challenge: trial evidence suggests they can be effective in reducing admissions and enhancing service user experience, but observational studies and audits indicate the benefits of CRT implementation on a national scale may be smaller and less consistent than originally expected [19, 20]. Means of supporting the effective implementation of the CRT model are therefore of high interest to service planners. Through testing a theoretically underpinned, clearly defined, extensive package of implementation resources for CRTs, our trial can provide evidence to address this important issue.

### Trial status

The recruitment of service user and staff participants for outcome evaluation and the trial intervention are ongoing at the time of submission of this protocol for publication. The final report of the study to the funders is due for submission by April 2017.

## Additional files

Additional file 1: CORE CRT Service Improvement Programme Trial – Reporting Checklist. (DOCX 44 kb) Additional file 2: CORE CRT Service Improvement Programme – Participant Consent Form. (DOCX 120 kb)

## Abbreviations

CORE: Crisis Team Optimisation and Relapse Prevention (aka The CORE Study)
CRT: crisis resolution team
EBP: evidence-based practices program
