# Supplementary material for: The CORE Service Improvement Programme for mental health crisis resolution teams: study protocol for a cluster-randomised controlled trial
Source: Trials. 2016 Mar 22;17:158. doi: 10.1186/s13063-016-1283-7 (PMC4804533; doi:10.1186/s13063-016-1283-7)
Supplement: Additional file 2: — CORE CRT Service Improvement Programme – Participant Consent Form. (DOCX 120 kb) [file 13063_2016_1283_MOESM2_ESM.docx]

**Additional File 2 – CORE CRT Service Improvement Programme participant consent form**


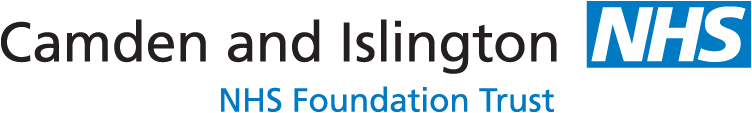


**Consent form for participation in service user interviews**

Version 1: 20.11.13

**Study Title: CORE Phase 4: Evaluation of implementation of a CRT Resource Kit**

Principal Investigator: Professor Sonia Johnson, UCL.

Research Worker:

1. I have read and understood the study information sheet dated 20.11.13.
2. I have had the opportunity to ask questions about the study.
3. I understand that my participation is voluntary and that I can withdraw at any time, without giving any reason, without my care being affected.
4. I understand that the Crisis Resolution Team (CRT) which has supported me me will know a researcher has asked me about taking part in the study, but that

anything I tell researchers will remain confidential unless it raises significant concerns about my own or someone else’s safety.

1. I consent to a researcher contacting me to arrange a research interview for the study.
2. I consent to my questionnaire responses being copied to an electronic database and written and electronic data being stored securely at University College London.
3. I understand that I will be given £10 as a gift in cash or as an Amazon voucher for my participation in this study once I have taken part in it.

1. If I choose to complete this questionnaire as a telephone interview, I agree for the study researcher to audio- record verbal confirmation of my consent to take part.
2. I consent to the interview with me being tape recorded
3. I agree to take part in the study.

Agreed way of completing the interview

Meeting the researcher in person

Completing the questionnaires by email

Completing the questionnaires by online survey

Phone interview with a researcher

Agreed way of receiving £10 gift of thanks for participating in the study

Accepting £10 in cash following a face-to-face interview

Receiving a £10 Amazon voucher by email

Delivery of a £10 in cash or Amazon voucher by a study researcher after the interview

I would like a copy of a report with the study findings when the study is over:

Yes

No

Preferred contact details:

Name:

Address:

Phone number(s):

E-mail address:

_____________________ _______________ _____________________

Name of participant Date Signature

___________________ _____________ ___________________

Name of Researcher Date Signature

Researcher use only:

Participant’s consent is recorded as:

1. Signed consent form, stored at UCL or UWE

2. Participant-completed (unsigned) consent form + accompanying email (stored at UCL or UWE)

3. Researcher-completed (unsigned) consent form + audio-recording of verbal consent (stored at UCL or UWE)

*(please tick one option)*
